# Supplementary figures and images for: Multichannel Silicon Probes for Awake Hippocampal Recordings in Large Animals
Source: Front Neurosci. 2019 Apr 26;13:397. doi: 10.3389/fnins.2019.00397 (PMC6497800; doi:10.3389/fnins.2019.00397)

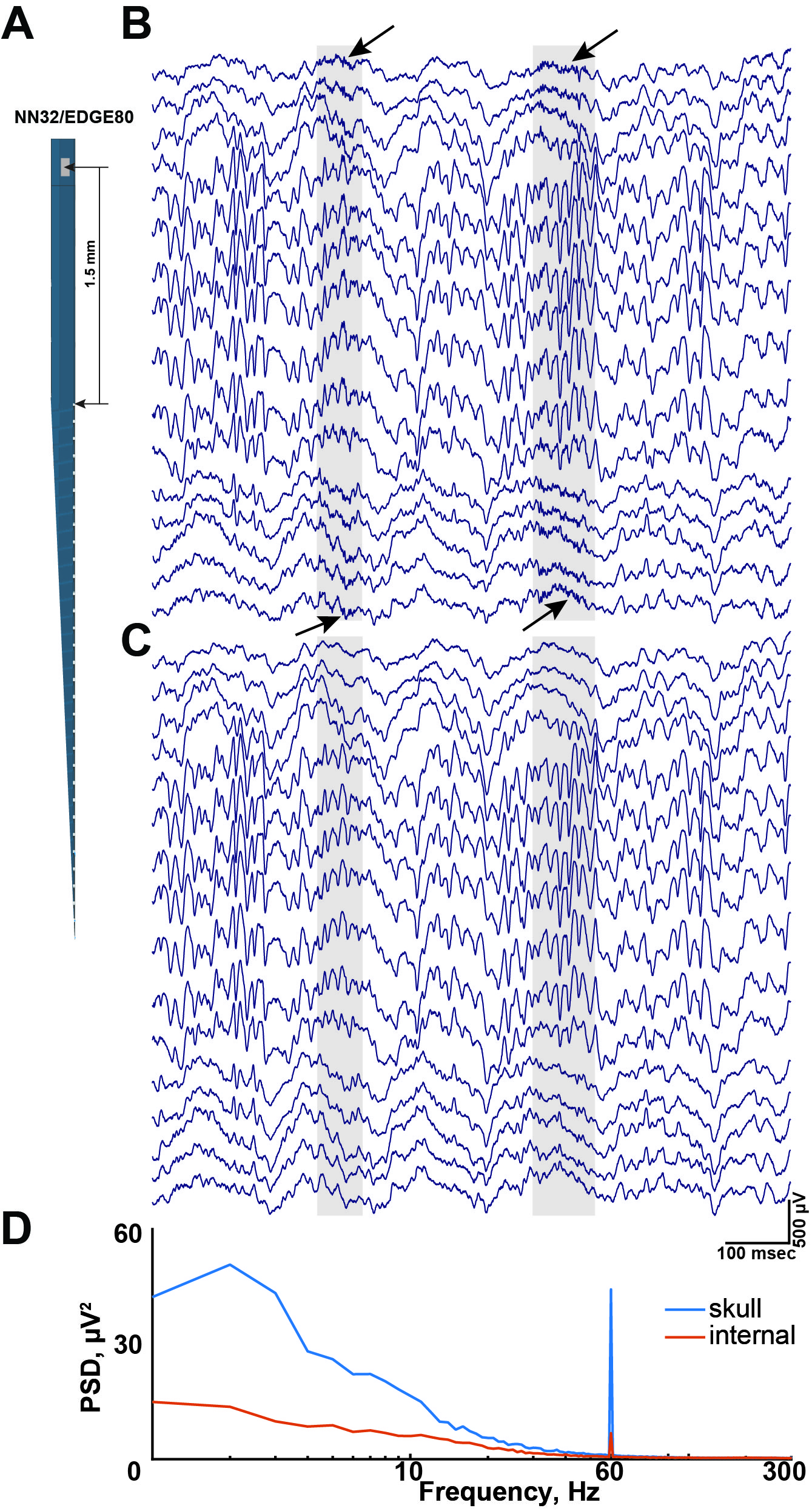

Supplement: FIGURE S1 — Internal reference on multichannel silicon probes reduces artifacts during chronic and acute recordings. (A) All multichannel silicon probes used in the study were custom-designed to have a top electrode site substituted for a low-impedance reference site (site area = 4,200 μm2) placed 1–2 mm above the most-proximal probe site. Schematic diagram of a NN32/EDGE80 silicon probe shows the location of internal reference. (B,C) During awake behaving recordings in pigs, the internal reference eliminated movement-associated artifacts. The same 1 s recording segment is shown referenced to a skull screw (B) vs. internal reference (C). (B) The highlighted areas (gray) show “movement artifacts” detected on all 31 channels during awake recordings (arrows). Note that only 16 out of 31 channels are displayed. (C) The noise associated with “movement artifacts” seen in (B) is eliminated after neurophysiological signals were re-referenced to the internal reference on the NN32/EDGE80 silicon probe. (D) Under the anesthetized preparation, the internal reference on the NN32/EDGE80 silicon probe eliminated most of the slow “drift” oscillations as well as the 60 Hz frequency peak, presumably from AC noise during acute recordings. [file Image_1.jpg]

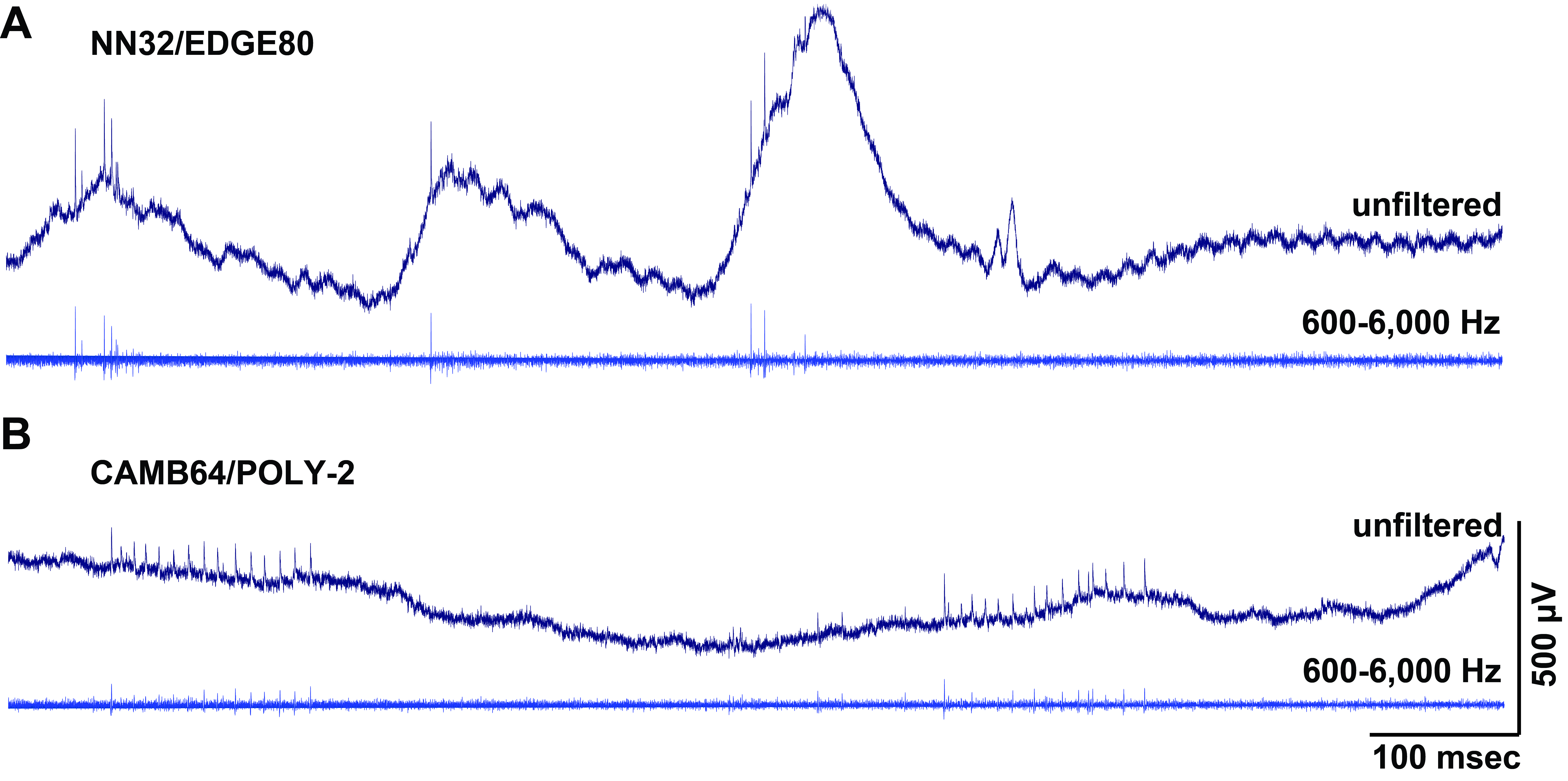

Supplement: FIGURE S2 — Multi-unit activity recorded with multichannel silicon probes designed for large animal electrophysiology. Recording profiles of multichannel silicon probes are displayed for NN32/EDGE80 and CAMB64/EDGE silicon probes. (A) Representative traces recorded from a single electrode site on NN32/EDGE80 silicon probe that was located in the pyramidal CA1 layer show raw, unfiltered signal (0.1–9,000 Hz, top trace) and the filtered signal used to identify spiking activity (600–6,000 Hz, bottom trace). Multiunit and oscillatory activity can be seen on both traces. (B) Representative traces recorded from a single electrode site on CAMB64/POLY-2 silicon probe that was located in the granular cell layer shows raw, unfiltered signal (0.1–9,000 Hz, top trace) and the filtered signal used to identify spiking activity (600–6,000 Hz, bottom trace). [file Image_2.JPEG]
